# Disease Mongering in Drug Promotion: Do Governments Have a Regulatory Role?

**DOI:** 10.1371/journal.pmed.0030198

**Published:** 2006-04-11

**Authors:** Barbara Mintzes

## Abstract

Most regulatory agencies, says Mintzes, fail to treat regulation of drug promotion as a public health concern. Unless this changes, she says, the public can expect more unfettered disease mongering.


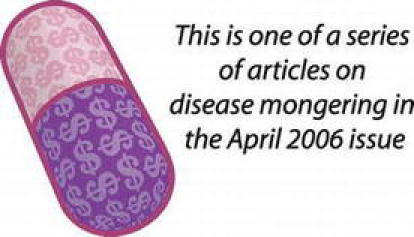


Ray Moynihan and colleagues describe disease mongering as, “widening the boundaries of treatable illness in order to expand markets for those who sell and deliver treatments” [ 1]. In this article, I examine one aspect of disease mongering: activities financed by drug companies to promote sales by expanding the pool of patients potentially treated by their products, when no benefit in terms of reduced morbidity is likely. New diseases may be “created” or existing conditions redefined. In theory, these activities are covered by national laws governing drug promotion that forbid misleading or deceptive advertising. However, enforcement is piecemeal and largely ineffective.

Drug regulation remains limited in many parts of the world. In 2004, fewer than one-sixth of countries had a well-developed system of drug regulation, and one-third had little to no regulatory capacity [ 2]. Although 89 countries (46%) reported active regulation of drug promotion, resources devoted to this work may be limited [ 3].

Full direct-to-consumer advertising (DTCA) of prescription drugs is legal in only the United States and New Zealand. However, in many other countries, unbranded disease-oriented advertising (in which no drug names are mentioned, but patients are often advised to “see your doctor”) is increasingly common. The Dutch Health-Care Inspectorate reviewed 28 product-specific marketing plans for prescription drugs, from ten companies, obtained through subpoenas from 1999 to 2002; 3.5% of their budgets were devoted to DTCA [ 4]. A market analyst reports that drug companies spent US$85 million on unbranded DTCA in Europe in 2004 [ 5]. Spending is expected to reach US$345.5 million by 2008. In 2005, the Australia–US free trade agreement allowed unbranded advertising in Australian media to be linked to branded information on Web sites [ 6]. Canada introduced more lenient policies on unbranded advertising in 1996, a shift that has occurred without legislative change [ 7].

A claimed benefit of disease-awareness campaigns is that the public becomes more aware of untreated health problems and seeks effective care at an earlier stage, leading to better health [ 8]. For this to happen, the campaigns must address important health concerns, focus on patients likely to benefit from diagnosis and treatment, and steer them towards appropriate care. For the individual patient, drug treatment is worth pursuing if potential benefits outweigh potential harm. But as healthier people are targeted, the added benefit of drug treatment can become increasingly elusive.

## Limited Regulatory Oversight of Unbranded Disease-Awareness Adverts

The US Food and Drug Administration (FDA) published a guidance in 2004 stating that unbranded adverts that are perceptually similar or otherwise linked to branded adverts are subject to FDA regulation, as are unbranded adverts by the manufacturer of the only drug in its class [ 9]. Otherwise, the FDA has no authority over the content of disease-oriented advertising, although it recommends responsible public health messages. The United Kingdom Medicines Health-Care Products Regulatory Agency has issued guidelines stating that the primary purpose of disease-awareness advertising must be health education on a disease and its management, not product promotion [ 10]. However, the Medicines Health-Care Products Regulatory Agency allowed Novartis' advertising on fungal nail infections (onychomycosis), which stressed high prevalence and infectiousness and guided viewers to prescription drugs, including Novartis' drug terbinafine (Lamisil) [ 10].

In the Netherlands, a similar Novartis campaign for onychomycosis prompted the Dutch government to take Novartis to court for illegal DTCA. The government lost the case as neither the product nor the manufacturer was named [ 11]. ‘t Jong and colleagues subsequently analyzed the effects of the campaign on primary care, using administrative data covering 150 practices. They studied the changes in rates of prescriptions of oral terbinafine (Lamisil) and itraconazole (Sporanox, a competitor to Lamisil), and the onychomycosis consultation rate, before and after the start of the campaign. Both onychomycosis consultations and prescriptions for terbinafine (Lamisil) grew, whereas prescriptions for the competitor drug declined [ 12]. Thus, an unbranded campaign had a brand-specific effect on sales, most likely because of concurrent branded promotion to physicians. ‘t Jong et al. noted the effects of promotion of a condition that is largely cosmetic (it usually causes no pain or suffering) on physicians' workload.

## Promoting Sales through Fear of Death

Pfizer, the manufacturer of Lipitor (atorvastatin), ran a campaign in France and Canada in 2003 with print adverts that used images of a tagged toe of a corpse (the Canadian campaign was in association with the Canadian Lipid Nurse Network and the Canadian Diabetes Association) ( Figure 1). On television, a youthful, healthy man died suddenly of a heart attack, leaving his family devastated with grief. The message of these two adverts was that cholesterol testing and treatment could prevent premature death from heart attacks in healthy people. This was at odds with existing scientific evidence: a 2003 meta-analysis of cholesterol-lowering drugs in primary prevention found no difference in mortality between drug and placebo [ 13].

**Figure 1:**
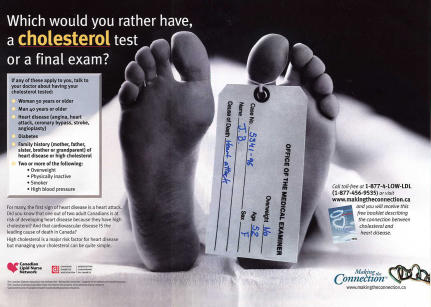
Disease-Awareness Campaign Sponsored by Pfizer, the Manufacturer of Lipitor (Image copyright: Pfizer Canada Inc./Making the Connection. All rights reserved.)

Jonathan Quick and colleagues at the World Health Organization raised concerns in the *Lancet* that the adverts misinformed the public about cardiovascular risks and could lead to harm from medically unjustified drug use [ 14]. They argued that governments should take a more active role in regulating disease-awareness campaigns to prevent misleading information from reaching the public. Complaints in Canada, which included the *Lancet* letter, resulted in no regulatory action [ 15]. A subsequent advert shows a man walking down a city street, unaware that he is about to be charged by a rhinoceros. The tagline is the following: “Living with high cholesterol, you never know what's around the corner.” The text stresses the risk of death from heart attacks. The only risk factor discussed is cholesterol.

Disease-awareness advertising is often the visible face of broader commercial influences. Eight of the nine authors of the US cholesterol treatment guidelines released in 2004 had financial links to manufacturers (Cleeman was the only member of the panel with no such ties; see http://www.nhlbi.nih.gov/guidelines/cholesterol/atp3upd04_disclose.htm) [ 16]. These guidelines extended treatment of high cholesterol to patient groups in which a morbidity and mortality advantage had not been established. A Pfizer financial report on atorvastatin (Lipitor) states, “There continues to be an opportunity for further growth of the cholesterol-lowering market….Evolving treatment guidelines continue to encourage the broad use of statin therapy” [ 17].

## Hormone Replacement Therapy and Menopause: An Ongoing Saga

Promotion of hormone replacement therapy (HRT) for disease prevention is a key example of disease mongering linked to drug sales. Women learned to view menopause in terms of increased health risks that could be prevented with HRT. The first long-term randomized controlled trial of HRT in healthy women, the Women's Health Initiative, found a 1% increase in absolute risks for serious harm over five years, mainly due to cardiovascular adverse effects [ 18]. The negative public health impact of HRT use by millions of women worldwide is likely to have been considerable. Regulatory agencies have changed labelling to warn potential users of serious risks and to advise limiting use to short-term symptomatic treatment, but have taken no broader action to review marketing of drugs for disease prevention.

On 28 December 2005, the first hit on a Google search on “menopause and estrogen deficiency” was a Merck Web site promoting an estrogen patch, and linking postmenopausal estrogen deficiency to reduced performance, fine motor skills, memory, and a reduction in “planned, targeted, flexible and adaptable thought” [ 19].

In 2006, a handbook for journalists, called *The Journalist's Menopause Handbook*, which was funded by Wyeth Canada and produced by a medical society (the Society of Obstetricians and Gynaecologists of Canada), fails to mention increased risks of strokes, heart attacks, pulmonary emboli, or symptoms of probable dementia associated with HRT [ 20]. The magnitude of breast cancer risk is described as no greater than lifestyle-associated risks. Hot flushes, mood and memory, appearance (wrinkles), sleep disturbances, bladder control, and sexual changes are listed as menopausal symptoms. Short-term HRT for moderate to severe symptoms is recommended as safe and effective, with “short-term” defined as up to five years [ 20]. Beyond the lack of established link between wrinkles and menopause (rather than ageing per se), is HRT really a reasonable treatment for wrinkles, given the cardiovascular, cancer, and dementia risks?

## Lower Thresholds for Symptomatic Treatment and Public Health

Mamdani and colleagues found that following the launch of celecoxib (Celebrex) and rofecoxib (Vioxx), more elderly patients in Ontario were treated with nonsteroidal anti-inflammatory drugs (NSAIDs) than previously [ 21]. The increase was attributable to use of Cox-2 inhibitors by people not previously taking NSAIDs. Paradoxically, although these newer drugs were promoted for greater gastrointestinal safety, Mamdani and colleagues found that approximately 650 more hospitalizations for gastrointestinal bleeds occurred per year after the drugs' introduction. In their conclusion, the authors stated the following: “Although we cannot prove causation, we believe that the striking temporal correlation, biological plausibility, and lack of any other trends that would explain the association strongly suggest that the two events are directly related” [ 21].

Another heavily promoted class of drugs are the proton pump inhibitors. Bashford and colleagues analyzed why patients were prescribed proton pump inhibitors during a five-year period in which prescribing increased 10-fold. By 1995, 46% of prescriptions were for off-label uses, mainly milder problems [ 22]. In 2004, researchers found a link between use of proton pump inhibitors and higher risks of Clostridium difficile infection in hospitalized patients [ 23]. A US magazine advertisement for esomeprazole (Nexium) in November 2005 (e.g., printed in *Family Circle*), a year after this study, warns readers that “something could be brewing” beneath their heartburn. A distressed woman is shown with a red scarf around her neck, and on the scarf is the following statement: “Behind this scarf acid could be burning the lining of her esophagus.” The advert quotes a high rate of erosive esophagitis among people with acid reflux, one in three, based on data on file at AstraZeneca. Although the advert contains the disclaimer that “only a doctor can determine if you have this condition,” the image of distress and the larger headlines—such as “Acid reflux disease can damage your esophagus” and “Nexium heals the damage”—convey the message to be anxious about heartburn and consider it a possible sign of more serious disease. Like many US adverts, this one offers a free trial.

## Questionable New Indications

Regulatory agencies have differed in their response to manufacturers' bids to market selective serotonin reuptake inhibitor (SSRI) antidepressants for “premenstrual dysphoric dysfunction” (PMDD). Business analysts linked the launch of the first drug in the US for this indication, fluoxetine (Sarafem), to Eli Lilly's pending loss of patent protection for Prozac (also fluoxetine) [ 24].

The European Medicines Evaluation Agency refused to approve drugs for PMDD, raising concerns that women “with less severe pre-menstrual symptoms might erroneously receive a diagnosis of PMDD resulting in widespread inappropriate short- and long-term use of fluoxetine” [ 25]. The US and Australia have approved SSRIs for PMDD, but Australia does not cover their costs [ 26].

Soon after Sarafem's launch, the FDA judged a TV advert to violate US law because it failed to distinguish clearly between PMDD and premenstrual syndrome [ 27]. A US community survey of women aged 14–24 found a 6% prevalence of PMDD. An additional 19% were “near-threshold” cases [ 28]. This survey likely overestimated PMDD, as classification was based on recall rather than daily symptom diaries (and most women were only mildly impaired), but the high “near-threshold” prevalence highlights the profitability of broadening diagnostic boundaries [ 29].

## Disease Claims in US DTCA: A Mixed Regulatory Response

Unlike many countries that rely primarily on industry self-regulation, the FDA regulates prescription drug promotion directly. Letters of violation to manufacturers are posted on the FDA Web site, with detailed rationales for regulatory decisions [ 30]. Of the 51 letters sent to companies in 2004 to mid-December 2005, 21 were either on DTCA exclusively ( *n* = 15) or on both DTCA and promotion for health professionals ( *n* = 6). For 15 out of 21 (71%) letters, reviewers from the FDA's Division of Drug Marketing, Advertising, and Communications raised concerns related to disease mongering ( Table 1). These concerns often consisted of (1) off-label promotion broadening approved indications and (2) misrepresentation of disease so as to exaggerate treatment effectiveness.

**Table 1:**
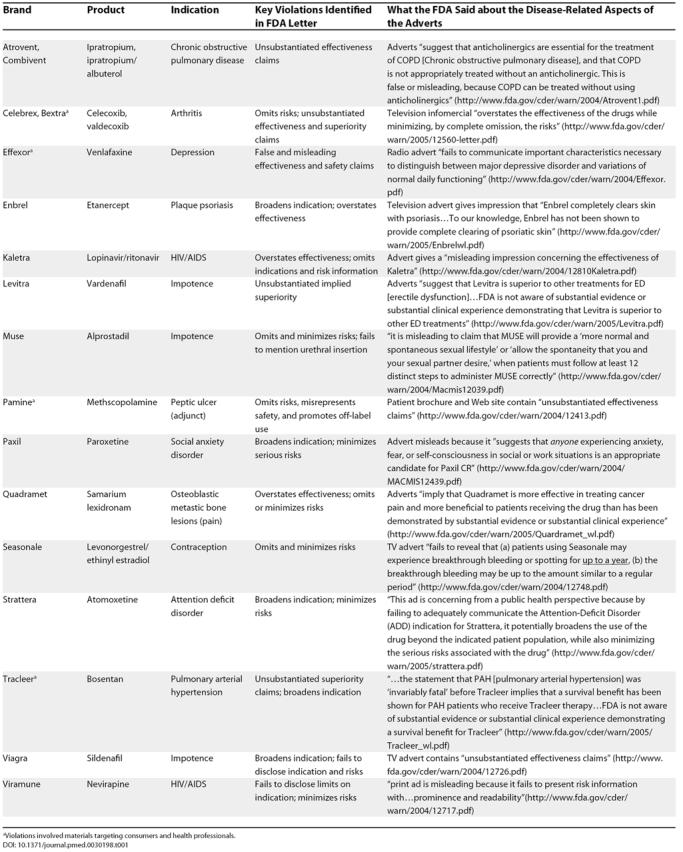
FDA Letters of Violation on Consumer-Directed Advertising, January 2004 to mid-December 2005

Many examples also exist of disease mongering in US DTCA that has not been subject to regulation. In a recent article in *PLoS Medicine*, Lacasse and Leo reviewed the evidence supporting the hypothesis that depression is caused by a serotonin deficiency, concluding that a lack of evidence exists to support this hypothesis [ 31]. They questioned the FDA's lack of attention to the claims in SSRI adverts for antidepressants that depression and anxiety disorders are caused by a chemical imbalance in the brain. The Irish regulatory agency has prohibited GlaxoSmithKline from making similar claims to support the use of paroxetine (Paxil) [ 32].

Kravitz and colleagues found more broadly that patient requests for advertised medicines could lead to off-label antidepressant prescribing for “adjustment disorder,” a disorder involving temporary distress due to a troubling life situation that rarely requires drug treatment [ 33]. Standardized patients received antidepressant prescriptions just over half the time if they requested the advertised antidepressant Paxil, whether they had symptoms of depression or adjustment disorder. If patients had not requested a drug, physicians were much less likely to prescribe antidepressants for adjustment disorder. This study provides experimental evidence of a link between patient requests for medicines and unnecessary medicalization.

## Conclusion: Is a More Robust Regulatory Response Needed?

Box 1 summarizes the types of disease-mongering activities companies can use to stimulate drug sales, including those described above.

The rationale for regulation of drug promotion is health protection, encouragement of appropriate medicine use, and prevention of deceptive advertising. The European community code on medicinal products for human use states that advertising of medicinal products “must encourage the rational use of the product and may not be misleading” [ 34]. Canada's Food and Drugs Act prohibits advertising of a drug that is “false, misleading or deceptive or is likely to create an erroneous impression regarding its character, value, quantity, merit or safety” [ 35]. The World Health Organization's Ethical Criteria for Medicinal Drug Promotion states that advertisements, “…should not take undue advantage of people's concern for their health” [ 36].

Disease mongering by definition creates erroneous impressions of the condition a product aims to treat and the merit and safety of treatment, and frequently provokes undue anxiety or exaggerates prevalence rates. Many of the activities in Box 1 are off-label promotions.

The prohibition of DTCA is consistent with regulatory aims to protect health and encourage appropriate medicine use. Unbranded disease-awareness campaigns for the condition a manufacturer's drug aims to treat are a form of DTCA. If these adverts are allowed under laws guaranteeing commercial freedom of expression, a regulatory rationale remains to (1) de-link them from suggestions to “ask your doctor” for a treatment and (2) to insist on prescreening of adverts by a government agency to ensure conformity with the law before they are broadcast or printed. Similarly, drug company funding of media promotions aiming to stimulate sales should be subject to the same regulatory control as direct advertising.

Better definitions are needed of the indications drugs are approved to treat, to ensure consistency with assessed outcomes in premarket trials. Evidence of benefit should be based on clinical outcomes, and greater caution is needed in introducing new diagnoses.

A key question is whether there is sufficient political will among government regulatory agencies to better enforce existing regulations governing drug promotion or to introduce new solutions. Most regulatory agencies fail to treat regulation of drug promotion as a public health concern. Unless this changes, the public can expect more unfettered disease mongering warning them that without the latest treatment, life will be grim indeed.

**Box 1. Forms of Disease Mongering Used to Expand Drug Sales**

- Promotion of anxiety about future ill-health in healthy individuals
- Inflated disease prevalence rates
- Promotion of aggressive drug treatment of milder symptoms and diseases
- Introduction of questionable new diagnoses—such as PMDD or social anxiety disorder—that are hard to distinguish from normal life
- Redefinition of diseases in terms of surrogate outcomes (i.e., osteoporosis becomes a disease of low bone density rather than fragility fractures)
- Promotion of drugs as a first-line solution for problems previously not considered medical, such as disruptive classroom behaviour or problematic sexual relationships.

## Abbreviations

DTCA: direct-to-consumer advertising
FDA: United States Food and Drug Administration
HRT: hormone replacement therapy
NSAID: nonsteroidal anti-inflammatory drug
PMDD: premenstrual dysphoric dysfunction
SSRI: selective serotonin reuptake inhibitor
